# Depression and Malnutrition for Prediction of Mortality after Transcatheter Aortic Valve Replacement: A Registry Study of a Tertiary Referral Hospital

**DOI:** 10.3390/diagnostics13152561

**Published:** 2023-08-01

**Authors:** Jolien Geers, Karen Van den Bussche, Bert Vandeloo, Dorien M. Kimenai, Ines Van Loo, Vincent Michiels, Daniele Plein, Stefan Beckers, Teun Muylle, Siddhartha Lieten, Bernard Cosyns, Nathalie Compté, Jean-François Argacha

**Affiliations:** 1 Department of Cardiology, Universitair Ziekenhuis Brussel, Vrije Universiteit Brussel (VUB), 1090 Brussels, Belgium; 2 BHF Centre for Cardiovascular Science, University of Edinburgh, Edinburgh EH9 3FB, UK; 3 Department of Cardiac Surgery, Universitair Ziekenhuis Brussel, Vrije Universiteit Brussel (VUB), 1090 Brussels, Belgium; 4 Department of Anesthesiology, Universitair Ziekenhuis Brussel, Vrije Universiteit Brussel (VUB), 1090 Brussels, Belgium; 5 Faculty of Medicine and Pharmacy, Vrije Universiteit Brussel (VUB), 1090 Brussels, Belgium; 6 Department of Geriatrics, Universitair Ziekenhuis Brussel, Vrije Universiteit Brussel (VUB), 1090 Brussels, Belgium; 7 Department of Geriatrics, CHU Ambroise Paré, 7000 Mons, Belgium

**Keywords:** frailty, depression, malnutrition, TAVR

## Abstract

Moderate to severe frailty is a predictor of a poor outcome after transcatheter aortic valve replacement (TAVR), but little is known about the prognostic importance of different geriatric frailty markers in an overall fit or pre-frail geriatric population undergoing TAVR. This retrospective study aimed to examine the incremental value of adding patient frailty markers to conventional surgical risk score to predict all-cause mortality in relatively fit elderly patients undergoing TAVR. Overall patient frailty was assessed using the comprehensive geriatric assessment frailty index (CGA-FI). Multivariable Cox regression models were used to evaluate relationships of different geriatric frailty markers with all-cause mortality and single and combined frailty models were compared to a baseline model that included EuroSCORE II factors. One hundred relatively fit geriatric patients (84 ± 4 years old, mean CGA-FI 0.14 ± 0.05) were included, and 28% died during a median follow-up of 24 months. After adjustment, risk of depression (geriatric depression scale 15 (GDS-15)) and malnutrition remained significantly associated with all-cause mortality (HR 4.381, 95% CI 1.787–10.743; *p* = 0.001 and HR 3.076, 95% CI 1.151–8.217; *p* = 0.025, respectively). A combined frailty marker model including both GDS-15 and malnutrition on top of EuroSCORE II improved the discriminative ability to predict all-cause mortality (change in c-index: + 0.044). Screening for those frailty markers on top of the traditionally used EuroSCORE II may improve risk stratification and prognosis in relatively fit geriatric patients undergoing TAVR.

## 1. Introduction

Aortic stenosis (AS) is the most common valvular heart disease in the western world, with a prevalence of up to 10% in those 80 years or older. It represents a major healthcare burden that is expected to further increase with improvements in life expectancy and an aging population [1,2]. When untreated, severe AS has a poor prognosis with a mortality of 50% within 2 years after the onset of symptoms [3].

TAVR has revolutionized the treatment of those patients and is nowadays not only used to treat severe AS patients at high surgical risk, but has been recognized as an alternative treatment strategy in patients at moderate and low risk. Given the huge burden of AS and the increasing number of patients that potentially fulfil the eligibility criteria for TAVR, the heart valve team faces different challenges regarding adequate indication and patient selection with obvious economic, clinical, and social implications.

Previous studies have shown that some patients do not have functional improvements or mortality benefit, and despite the emergence of data, it often remains difficult to determine factors of poor outcomes and survival after TAVR [4]. Conventional surgical risk models, such as the EuroSCORE II and Society of Thoracic Surgery predicted risk of mortality score (STS-PROM) [5,6,7] are traditionally applied to the TAVR population, but remain often suboptimal to predict outcomes after TAVR [8]. Indeed, TAVR is increasingly used in the elderly population, and some geriatric parameters affecting outcomes after TAVR may be missed by these scoring systems.

Frailty, a geriatric syndrome of decreased reserves that leads to vulnerability and an adverse outcome after an acute stressor [9], has a high prevalence in patients with AS, ranging from 26% up to 86%, and moderate to severe frailty in patients undergoing TAVR has been associated with adverse outcomes, both short- and long-term mortality, and increasing costs [10,11,12,13,14,15,16,17]. However, previous studies provided heterogeneous results because of the wide variety of domains that can be impacted by frailty (cognition, depression, nutrition, dependence, physical function), the use of different tests, and the lack of a clear and generally accepted frailty assessment. Furthermore, although moderate to severe frailty is a predictor of a poor outcome, little is known about the prognostic importance of different geriatric frailty markers in an overall fit or pre-frail population.

In the present study, we aimed to investigate the ability of different geriatric frailty markers to predict all-cause mortality in an overall fit or pre-frail elderly population of patients undergoing TAVR. We then wished to assess whether measuring those frailty markers adds incremental prognostic value to the traditionally used EuroSCORE II. 

## 2. Materials and Methods

### 2.1. Study Population

This is a retrospective single-center cohort study that included fit or pre-frail patients >75 years with severe and symptomatic AS who underwent TAVR at UZ Brussel, a university and tertiary referral hospital from Brussels, Belgium, between March 2015 and January 2020. Severe AS was defined as a calculated aortic valve area (AVA) < 1.0 cm^2^ or AVA indexed to body surface area (BSA) < 0.6 cm^2^/m^2^. AS was considered symptomatic if patient had dyspnea ≥2 based on the New York Heart Association (NYHA) classification. Patients who died within 30 days or during the index procedure hospitalization were excluded from the analysis since this reflects procedural mortality according to the Valve Academic Research Consortium (VARC)-2 definitions [18].

Overall patient frailty was estimated using the CGA frailty index (CGA-FI) score. This score was calculated using an online calculator [19] assessing 4 different items: (1) medical history addressing the presence of 21 comorbidities; (2) functional status based on the Katz score; (3) performance tests throughout the assessments of the MMSE, gait speed from a 4-m walk test, time to complete 5 chair stands, and dominant grip strength; and (4) nutritional status based on a BMI < 22 kg/m^3^ and serum albumin < 3.5 g/L. The calculated score ranged from 0 to 1 and categorized patients as non-frail (<0.20), pre-frail (0.20–0.35), and frail (>0.35) [16,20]. The study population consisted only of patients with a CGA-FI score below 0.35.

### 2.2. Ethics

The study was performed according to the ethical guidelines of the 1975 Declaration of Helsinki and approved by the Ethics Review Committee of UZ Brussel (B.U.N. 1432022000098).

### 2.3. Clinical Characteristics

We retrospectively extracted data from the electronic patient records through chart review. For all patients, information on age, sex, and body mass index (BMI) were retrieved. The baseline cardiovascular assessment included a patient history as well as a baseline heart failure assessment based on severity of symptoms using the NYHA functional classification. Information on relevant comorbidities was collected: presence of diabetes, chronic obstructive pulmonary disease (COPD), atrial fibrillation, hypertension, coronary artery disease (CAD), peripheral arterial disease, history of stroke or transient ischemic attack (TIA), and myocardial infarction (MI). A history of relevant procedures was also obtained: percutaneous coronary intervention, coronary artery bypass grafting, and sternotomy. Data on medication use (angiotensin converting enzyme (ACE) inhibitors or angiotensin II receptor blockers (ARB), adenosine diphosphate (ADP) inhibitors, and antidepressants), laboratory values (estimated glomerular filtration rate (eGFR), creatinine, white blood cell count (WBC), hemoglobin, hematocrit and platelet count), cardiologic and hemodynamic investigations (electrocardiography, echocardiography, coronary angiogram and right heart catheterization) were also collected. For all patients, a baseline EuroSCORE II and STS-PROM cardiac surgery risk score were calculated before the procedure when patients were screened in aortic valve clinics or inpatient wards [21,22].

### 2.4. Procedural and Hospitalization-Related Characteristics

The following procedural characteristics were recorded: type of anesthesia (full anesthesia or conscious sedation), valve type (Evolute (Medtronic^®^) or Accurate Neo (Boston^®^)), valve size, approach type (transfemoral or non-transfemoral), and pre- and post-valve implantation balloon dilatation. The measured hospitalization-related characteristics were length of stay, presence of major adverse cardiovascular event, transfusion, the presence of at least moderate aortic and mitral regurgitation post-TAVR procedure, and pacemaker implantation.

### 2.5. Geriatric Frailty Markers

A comprehensive geriatric assessment (CGA) before the procedure was performed by a multidisciplinary team (geriatrician, ergo therapist, physiotherapist, nurse, psychologist) in every patient entering this study. This assessment was mandatory for procedure reimbursement. The CGA included a structured interview, a physical examination, a functional assessment, and blood sampling.

Geriatric parameters were measured using the following validated instruments: *Functional status* was assessed using the Katz index of independence in activities of daily living (ADL), which comprises 6 items (bathing, dressing, toileting, transferring, continence, and feeding) with a total score of 24 [23]. *Cognitive function* was screened using the Mini Mental State Examination (MMSE) questionnaire (30 items, scored between 0 (worst) and 30 (best)) [24]. *Polypharmacy* was defined as the use of five or more medications a day. *Malnutrition* was defined as a BMI lower than 22 kg/m^2^ or albumin lower than 3.5 g/L [25,26]. *The probability of having a depressed mood* was assessed by the geriatric depression scale 15 (GDS-15) [27]. A score ≥5 out of 15 was considered as a higher risk to develop a depression in the near future [27]. *Grip strength* was assessed in the participant’s dominant hand using a dynamometer with the elbow in 90-degree flexion in standing position [28]. *Gait speed* was assessed using the 4 m-walk test, and slower than 0.8 m/sec was considered a marker of frailty [29]. *The chair stand test* (CST) measured how long it took to perform five consecutive chair-stands (timed to 0.1 s) from a seated position on a 45 cm tall chair, with arms folded across the chest, and a time longer than 14 s was considered as a lack of strength [30]. The *short physical performance battery* (SPPB) combines the previous two tests complemented with three increasingly more difficult standing balance tests, and a score <10 was considered as a risk of mobility-related disability [31]. The *fall risk* was evaluated by the Tinetti performance-oriented mobility assessment and timed up and go (TUG) test. The Tinetti test is the most widely used clinical test to assess a person’s static balance ability and gait, and a score of <20 was rated as an impairment [32]. TUG involves the participant rising from a standard armchair, walking a distance of 3 m at a normal and safe pace, turning around, and walking back to the chair and sitting down again, and a TUG ≥ 20 s corresponds to an increased risk of fall [33]. *Comorbidities* were estimated using the Charlson comorbidity index (CCI), which consists of 17 items adjusted for age, with a score of 0 if there are no comorbidities present [34].

Furthermore, there was a routine clinical follow-up scheduled 1, 3, 6, 12, and 24 months after the TAVR procedure. Some patients received also a dedicated geriatric follow-up in the first-year post-TAVR procedure and were included in a geriatric care program. This geriatric follow-up was recommended but not mandatory, and left to the patient’s and general practitioner’s decision. If patients did not attend scheduled cardiologist or geriatrician consultations, they were further contacted by phone or mail by a study nurse. If unsuccessful, relatives and/or family physicians were contacted.

### 2.6. Study Endpoint

The clinical endpoint of this study comprised death from any cause during follow-up. Post-procedural related mortality was excluded by examining only events which occurred beyond 30 days of TAVR.

### 2.7. Statistical Analysis

Continuous variables are presented as mean with standard deviation (SD) or median with 25th to 75th percentile, as appropriate. Categorical variables are presented as absolute numbers (%). Comparisons are made between those who did and did not die during follow-up. For continuous variables, between-group differences were assessed using an independent-samples Student’s *t*-test for normally distributed data, and the Mann–Whitney U-test for non-normally distributed data. For categorical data, the chi-square (χ^2^) test was used to evaluate differences between groups.

Kaplan–Meier curves were constructed to evaluate the relationship between geriatric frailty measures and all-cause death. Univariable Cox proportional hazards regression analysis was conducted to assess the relationship of different frailty markers and all-cause mortality. Frailty markers which showed a significant relationship with all-cause mortality in univariable models were further assessed in multivariable Cox regression models. We assessed these geriatric frailty markers in multivariable models that (1) included sex and age, and (2) included EuroSCORE II as covariate(s) in the model. Frailty scales were primarily analyzed in their continuous form and secondarily in their dichotomous form based on a priori cutoffs. Accordingly, we evaluated discrimination for single and combined frailty marker models using Harrell’s c-statistic. Statistical analysis was performed in SPSS Statistic for Windows, version 28.0 (IBM), and using R, version 3.6.2. A *p* value < 0.05 was considered significant.

## 3. Results

### 3.1. Baseline Characteristics of the Study Population and Clinical Endpoint Groups

The study population consisted of 100 patients (39% male, mean age 84 ± 4 years old) who received a TAVR procedure between 2015 and 2020 (Supplemental Figure S1). During the median follow-up period of 24 [20,21,22,23,24,25,26] months, all-cause death occurred in 28% of the study population. Clinical, hemodynamic, laboratory, and procedural characteristics of the overall study population and the primary endpoint groups are summarized in Table 1. Patients who reached the clinical endpoint had a significantly higher left atrium volume index (LAVI) and a lower albumin level at baseline evaluation (57.5 ± 17.2 mL/m^2^ vs. 48.7 ± 14.5 mL/m^2^; *p* = 0.042 and 37.8 ± 4.4 g/L vs. 40.4 ± 5.8 g/L; *p* = 0.046, respectively). The median EuroSCORE II in the overall population was 4.6 [2.8–7.7] and was significantly higher in patients who died during follow-up compared to those who survived (6.1 [3.7–10.4] vs. 4.0 [2.7–6.0]; *p* = 0.025), as opposed to the STS-PROM score which did not differ between groups (3.6 [2.5–5.0] vs. 4.1 [2.7–5.0]; *p* = 0.539). A lower percentage of patients who died during follow-up were included in a geriatric care program and received a dedicated geriatric follow-up in the first year after TAVR, although this trend did not reach statistical significance (21% in the primary endpoint group vs. 39% in the group without a primary endpoint; *p* = 0.098).

### 3.2. Geriatric Frailty Characteristics in Relation to Clinical Endpoint

The mean CGA-FI score of the overall study population was 0.14 ± 0.05 with 14% considered as pre-frail with a score between 0.20–0.35. With the exception of GDS-15 and malnutrition, no statistically significant differences in frailty markers were observed between those who did and did not die during follow-up (Table 2). 

**Figure 1:**
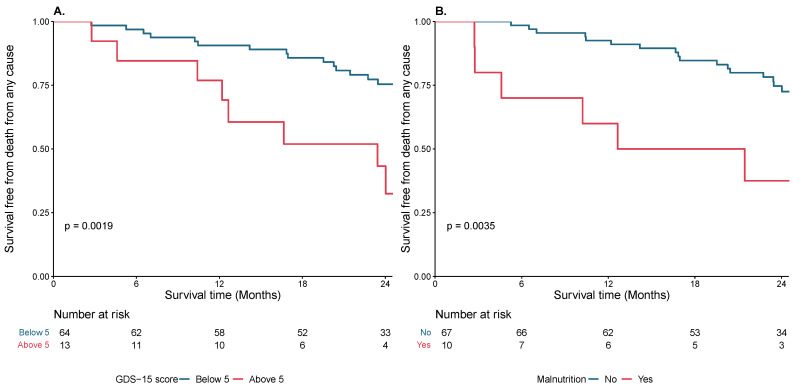
Kaplan–Meier curves with log-rank for overall survival of patients treated with TAVR according to (**A**) geriatric depression scale 15 and (**B**) malnutrition status.

A significantly higher percentage of patients who reached the clinical endpoint were at risk for depression as determined by a GDS-15 score ≥ 5 (38% vs. 13%; *p* = 0.011) (Figure 1A). Patients with a higher probability of having a depressed mood had more frequently a gait speed slower than 0.8 m/s (80% vs. 34%; *p* = 0.006), presented with lower TAPSE (16.5 ± 5.7 vs. 20.0 ± 4.9; *p* = 0.042) and LVEF (50 [44–56] vs. 55 [50–60]; *p* = 0.041), and had a longer hospitalization duration (10 [9–20] vs. 8 [6–12]; *p* = 0.004) (Supplemental Table S1).

Malnutrition was present in 14% of the overall population with a significantly higher proportion in the group of patients with clinical endpoint as compared to those without (28% vs. 7%; *p* = 0.013) (Figure 1B). Patients with a positive malnutrition screening presented with a higher LAVI (60 ± 22 vs. 48 ± 13; *p* = 0.034) and lower LVEF (50 [30–55] vs. 55 [50–60]; *p* = 0.049), and had a longer hospital duration (15 [7–27] vs. 8 [6–12]; *p* = 0.020) (Supplemental Table S2).

### 3.3. Frailty Marker Risk Models to Predict All-Cause Mortality

All frailty markers were tested in univariable Cox regression analysis, but only baseline GDS-15 score and malnutrition status were significantly associated with all-cause mortality (HR 3.598, 95% CI 1.519–8.521; *p* = 0.004 and HR 3.672, 95% CI 1.443–9.345; *p* = 0.006, respectively; Table 3 and Table S3). The predictive ability of both frailty markers for all-cause mortality remained significant after adjustment for age and sex (*p* < 0.001 for GDS-15 and *p* = 0.007 for malnutrition status; Table 3) and after adjustment for EuroSCORE II (*p* = 0.002 for GDS-15 and *p* = 0.033 for malnutrition status; Table 3). In contrast, no differences were found in patients who did and did not enter a post-TAVR geriatric care program (*p* = 0.079, Supplemental Figure S2).

Accordingly, we evaluated whether GDS-15 and malnutrition would improve discriminative performance as compared with the base model using EuroSCORE II. The c-index of the base model was 0.702, a GDS-15 score ≥ 5 improved the c-index by +0.025, and malnutrition improved the c-index by +0.039 (Table 4). A combined risk model that included both EuroSCORE II, GDS-15, and malnutrition further improved the c-index by +0.044 (Table 4). 

## 4. Discussion

The aim of the present study was to determine to ability of different patient frailty characteristics to predict all-cause mortality in overall fit or pre-frail elderly patients undergoing TAVR. We found that both GDS-15 score ≥ 5, indicating a high risk to develop a depressed mood, and malnutrition status were strong predictors for all-cause mortality after TAVR. Moreover, measuring those frailty markers adds incremental value to traditional risk models, such as the traditionally used EuroSCORE II, to predict all-cause mortality after TAVR. We did not observe a significant relationship between other evaluated geriatric frailty markers and all-cause death. Altogether, our study indicates that a risk assessment strategy that includes depression and malnutrition status on top of EuroSCORE II may contribute to further improvement in the prediction of worse outcomes in overall fit geriatric patients who undergo TAVR.

Depression, along with chronic kidney diseases, anemia, pulmonary disease, and cardiovascular disease is one of the most common diseases in frail people and is highly prevalent among patients with AS undergoing AVR [35]. This is not only the case for frail AS patients as 20% of our relatively fit geriatric population was identified as being at risk of developing depression. We found a significant association between baseline depression risk and all-cause mortality after TAVR, with 38% of deaths observed in the group with a GDS-15 score ≥ 5 compared to 13% of deaths in those with a GDS-15 score < 5. This is an interesting finding as depression is rarely a component of existing frailty calculators and not integrated in a standard pre-TAVR workup. Similarly, in a prospective multicenter cohort of 1035 patients undergoing TAVR and SAVR, 31.5% of patients had positive results of screening for depression, and the risk of depression was significantly associated with 1-month and 12-month mortality [36]. In that study, all patients with depression were re-evaluated at 6 months after AVR allowing them to report that the highest risk group consisted of patients with persistent depression after 6 months [36]. Given its high prevalence in cardiovascular patients and its association with unfavorable outcomes in TAVR patients, our findings support the systematic integration of a depression risk evaluation throughout GDS-15 assessment in the process of selecting candidate patients for TAVR. This approach has the potential to improve prognostic evaluation and to identify patients who may benefit from further psychiatric evaluation.

Another important finding of our study was the significant association between malnutrition and all-cause mortality after TAVR. Poor nutritional status is common in a geriatric population, hospitalized patients, and patients with cardiovascular disease, and is an independent risk factor for low immune response, poor clinical outcome, and long-term mortality [37,38]. The relationship between nutritional status and morbidity and mortality among AS patients treated with TAVR has already been demonstrated in several studies [15,17,39,40]. A recent study demonstrated that baseline hypoalbuminemia (<3.5 g/L) was associated with an increased mortality at 3 years, even after adjustment for frailty, sex, STS-PROM score, liver disease, acute kidney injury, and hospital readmission [41]. The results of these studies together with our findings suggest that pre-TAVR interventions aimed at improving nutritional status could improve patients’ prognosis, and that screening for malnutrition should be systematically included in the pre-TAVI workup.

Predicting mortality after TAVR in this elderly population remains a difficult challenge. The most recent ESC guidelines, published in 2021, recommend systematic assessment of frailty before TAVR using an objective assessment rather than a subjective approach, such as the “eyeball” test [42]. However, frailty covers a broad range of domains and ESC guidelines do not specify which parameters should be included in day-to-day clinical practice given the existing uncertainty whether the effort of measuring such scales is justified by meaningful improvements in discrimination. In contrast to previously published studies, CGA-FI, a recently developed score to ensure a standardized evaluation of overall patient frailty, was not an independent risk predictor for all-cause mortality in our TAVR population. An important remark and possible explanation is that our study population consisted of relatively fit geriatric patients as opposed to referent studies [10,11,16,43]. The Belgian health insurance imposes strict criteria to obtain procedure reimbursement, which has led to the rejection of too frail patients presenting co-morbidities not compatible with reimbursement regulations. The mean CGA-FI score of our study population was 0.14 with only 14% of patients who were classified as pre-frail with a score between 0.20–0.35, and no patients who were classified as frail (score > 0.35). This may explain the discrepancy of our results compared to other studies and suggests that the use of the CGA frailty index to predict mortality after TAVR should be reserved for more frail patients. In our relatively fit geriatric population, we showed that adding both depression risk and malnutrition evaluation to the baseline assessment of TAVR patients using EuroSCORE II resulted in the most robust model to predict outcomes, leading to the largest c-statistic improvement of 0.044. This clinically meaningful improvement in discrimination of patient outcomes after TAVR can further justify the integration of those frailty scales in day-to-day clinical practice.

### Limitations

Limitations associated with the retrospective nature, lack of sample size calculation, being a single-center study, and relatively small sample size need to be taken into account. First, our study was likely underpowered to observe the effect of participation in a geriatric care program on long-term mortality after TAVR. A systematic review did find that a rehabilitation program in TAVR improves functional capacity and quality of life in a similar manner to SAVR patients, suggesting this may also impact other clinical endpoints [44]. Second, the GDS-15 score is a validated instrument to screen for depression, but there was no routine psychiatric evaluation to confirm the actual diagnosis of depression. Similarly, in the case of malnutrition, several studies highlighted the shortcomings of using only albumin and BMI as markers for malnutrition. Ideally, a nutritional risk assessment should include medical history, current and past dietary intake, medications, laboratory values, and anthropometric measurements [45]. However, it is not always possible to obtain all those variables, and by using albumin and BMI only, important information about nutrition status is gathered in an easy and straightforward way. Third, the predictive ability of GDS-15 and malnutrition needs to be validated in an external cohort of TAVR patients. Furthermore, the data reflect the practice at the earlier period of TAVR procedures at the center of inclusion with high levels of general anesthesia, surgical cut-down to access the femoral artery, a high rate of pacemaker implantation, and a relatively long hospital stay. Nowadays, the procedure is routinely performed with full percutaneous approach and local sedation, significantly reducing overall hospital time. 

## 5. Conclusions

In our relatively fit geriatric population of aortic stenosis patients undergoing TAVR, both risk of depression and malnutrition are prevalent and are associated with higher risk of all-cause mortality at two-year follow-up. Measuring these frailty markers could add incremental value above the existing risk model EuroSCORE II to predict all-cause mortality after TAVR, and screening for risk of depression and malnutrition may, therefore, improve risk stratification and prognosis in TAVR patients.

## Figures and Tables

**Table 1:** Baseline clinical, hemodynamic, laboratory, and procedural characteristics of the overall study population and the primary endpoint groups.

	Overall Population (*n* = 100)	Primary Endpoint (*n* = 28)	No Primary Endpoint (*n* = 72)	*p*-Value
*Clinical*				
Male, *n* (%)	39 (39)	14 (50)	25 (35)	0.160
Age (years)	84 ± 4	84 ± 4	84 ± 4	0.979
Body mass index (kg/m^2^)	27.5 ± 4.9	27.3 ± 4.8	27.6 ± 5.0	0.793
Hypertension, *n* (%)	78 (78)	22 (79)	56 (78)	0.931
Diabetes mellitus, *n* (%)	24 (24)	8 (29)	16 (22)	0.504
Atrial fibrillation, *n* (%)	33 (33)	8 (29)	25 (35)	0.557
Prior MI, *n* (%)	15 (15)	4 (14)	11 (15)	0.901
CAD, *n* (%)	42 (42)	12 (43)	30 (42)	0.914
Prior stroke or TIA, *n* (%)	9 (9)	4 (14)	5 (7)	0.249
Sternotomy, *n* (%)	24 (24)	7 (25)	17 (24)	0.884
COPD, *n* (%)	19 (19)	5 (18)	14 (19)	0.856
EuroSCORE II	4.6 [2.8–7.7]	6.1 [3.7–10.4]	4.0 [2.7–6.0]	0.025
STS-PROM	3.9 [2.6–5.0]	3.6 [2.5–5.0]	4.1 [2.7–5.0]	0.539
*Medication, n (%)*				
ADP inhibitor	7 (7)	2 (7)	5 (7)	0.972
ACE/ARB inhibitor	57 (57)	18 (64)	39 (54)	0.359
Antidepressants	8 (8)	2 (7)	6 (8)	0.844
*Hemodynamic and laboratory*				
AVA (cm^2^)	0.76 ± 0.22	0.74 ± 0.16	0.76 ± 0.24	0.654
PG (mmHg)	71.3 ± 27.8	68.9 ± 19.4	72.2 ± 30.5	0.593
LVEF (%)	55 [50–60]	50 [50–59]	55 [50–60]	0.326
TAPSE (mm)	19.7 ± 5.3	19.1 ± 5.9	19.9 ± 5.1	0.604
SPAP (mmHg)	42.0 ± 15.3	45.4 ± 21.1	40.5 ± 11.8	0.279
LAVI (mL/m^2^)	50.9 ± 15.6	57.5 ± 17.2	48.7 ± 14.5	0.042
eGFR (mL/min/1.73 m^2^)	53.2 ± 14.3	49.4 ± 14.6	54.6 ± 13.9	0.100
Hematocrit (%)	34.5 ± 11.5	33.3 ± 11.8	35.0 ± 11.5	0.509
WBC (×10^3^/mm^3^)	7.0 [5.6–8.1]	7.1 [5.2–8.0]	7.0 [5.7–8.2]	0.514
Albumin (g/L)	39.6 ± 5.5	37.8 ± 4.4	40.4 ± 5.8	0.046
*Procedural and hospitalization*				
Full anesthesia, *n* (%)	75 (75)	22 (79)	53 (74)	0.607
TF approach, *n* (%)	97 (97)	27 (96)	70 (97)	0.835
Length of stay (days)	8 [6–12]	10 [6–23]	8 [6–12]	0.263
Pacemaker, *n* (%)	18 (18)	6 (21)	12 (17)	0.578
MR ≥ 2/4, *n* (%)	29 (29)	10 (36)	19 (26)	0.356
AR ≥ 2/4, *n* (%)	10 (10)	2 (8)	8 (12)	0.581
Geriatric follow-up, *n* (%)	34 (34)	6 (21)	28 (39)	0.098

Data are presented as number (percent), mean ± standard deviation if normally distributed or median (interquartile range) if not normally distributed. MI: myocardial infarction; CAD: coronary artery disease; TIA: transient ischemic attack; COPD: chronic obstructive pulmonary disease; STS-PROM: Society of Thoracic Surgery predicted risk of mortality score, ADP: adenosine diphosphate; ACE: angiotensin-converting enzyme; ARB: angiotensin receptor blocker; AVA: aortic valve area; PG: peak gradient; LVEF: left ventricular ejection fraction; TAPSE: tricuspid annular plane systolic excursion; SPAP: systolic pulmonary artery pressure; LAVI: left atrium volume index; eGFR: estimated glomerular filtration rate; WBC: white blood cell count; TF: transfemoral; MR: mitral regurgitation; AR: aortic regurgitation.

**Table 2:** Baseline geriatric characteristics of the overall study population and the primary endpoint groups.

	Overall Population	Primary Endpoint	No Primary Endpoint	*p*-Value
Katz (*n* = 97)	7 [6–8]	7 [6–8]	7 [6–8]	0.818
MMSE (*n* = 97)	27 [25–29]	27 [25–29]	27 [24–29]	0.420
Polypharmacy (*n* = 98)				
≥5 medicines	90 (92)	24 (89)	66 (93)	0.511
Timed up and go (*n* = 93)	15.4 ± 9.8	13.7 ± 6.9	16.1 ± 10.6	0.305
≥20 s	18 (19)	5 (20)	13 (19)	0.924
Chair stand test (*n* = 90)	15.9 ± 9.6	14.2 ± 8.7	16.4 ± 9.9	0.347
≥14 s	66 (73)	16 (70)	50 (75)	0.636
Gait speed (*n* = 75)	0.83 ± 0.26	0.80 ± 0.33	0.84 ± 0.24	0.393
≤0.8 m/s	30 (40)	7 (39)	23 (40)	0.912
Tinetti (*n* = 81)	25 [21–27]	24 [20–27]	26 [21–27]	0.711
<20	16 (20)	5 (23)	11 (19)	0.681
SPPB (*n* = 80)	7.7 ± 2.8	8.2 ± 2.4	7.6 ± 3.0	0.393
<10	54 (68)	14 (70)	40 (67)	0.784
Grip strength (*n* = 68)	42.5 ± 16.4	46.8 ± 14.8	41.2 ± 16.8	0.235
GDS-15 (*n* = 91)	1 [1–4]	3.5 [1–5]	1 [0–3]	0.045
≥5	18 (20)	9 (38)	9 (13)	0.011
Malnutrition (*n* = 80)				
BMI < 22 or albumin < 3.5 g/L	11 (14)	7 (28)	4 (7)	0.013
BMI < 22	3 (3)	2 (7)	1 (1)	0.063
Albumin < 3.5 g/L	11 (14)	7 (28)	4 (6)	0.002
CCI (*n* = 100)	5.0 ± 1.1	5.1 ± 1.2	4.9 ± 1.1	0.399
CGA-FI (*n* = 100)	0.14 ± 0.05	0.15 ± 0.06	0.14 ± 0.05	0.200
>0.2	14 (14)	5 (18)	9 (12)	0.488

Data are presented as number (percent), mean ± standard deviation if normally distributed or median (interquartile range) if not normally distributed. MMSE: mini mental state examination; SPPB: short physical performance battery; kPa: kilopascal; GDS-15: geriatric depression scale 15; BMI: body mass index; CCI: Charlson comorbidity index; CGA-FI: comprehensive geriatric assessment frailty index.

**Table 3:** Univariable and multivariable Cox regression analysis of GDS-15 and malnutrition for the prediction of all-cause mortality.

	HR	95% CI	*p*-Value
Unadjusted			
GDS-15 ≥ 5	3.60	1.52–8.52	**0.004**
Malnutrition	3.67	1.44–9.34	**0.006**
Adjusted for age and sex			
GDS-15 ≥ 5	5.07	2.00–12.87	**<0.001**
Malnutrition	3.83	1.45–10.12	**0.007**
Adjusted for EuroSCORE II			
GDS-15 ≥ 5	4.15	1.72–10.01	**0.002**
Malnutrition	2.89	1.09–7.67	**0.033**
Combined model with EuroSCORE II			
GDS-15 ≥ 5	4.38	1.79–10.74	**0.001**
Malnutrition	3.08	1.15–8.22	**0.025**

HR: hazard ratio; CI: confidence interval; GDS-15: geriatric depression scale 15.

**Table 4:** C-index of the single and combined frailty marker models compared to a baseline model including EuroSCORE II.

	C-Index	ΔC-Index
Unadjusted		
GDS-15	0.628	
Malnutrition	0.593	
EuroSCORE II	0.702	
Adjusted model		
EuroSCORE II + GDS-15	0.727	0.025
EuroSCORE II + Malnutrition	0.741	0.039
EuroSCORE II + GDS-15 + Malnutrition	0.746	0.044

GDS-15: geriatric depression scale 15.

## Data Availability

Data sharing is not applicable to this article.

## Supplementary Materials

The following supporting information can be downloaded at: https://www.mdpi.com/article/10.3390/diagnostics13152561/s1, Figure S1: Flow chart of study population after applying exclusion and inclusion criteria; Figure S2: Kaplan–Meier curves with log-rank test for overall survival of patients treated with TAVR according to inclusion in post-TAVR geriatric care program; Table S1: Baseline characteristics according to depression status; Table S2: Baseline characteristics according to malnutrition status; Table S3: Univariable Cox regression analysis of frailty markers for all-cause mortality prediction.

## References

[1] Osnabrugge R.L., Mylotte D., Head S.J., Van Mieghem N.M., Nkomo V.T., LeReun C.M., Bogers A.J., Piazza N., Kappetein A.P. (2013). Aortic Stenosis in the Elderly: Disease Prevalence and Number of Candidates for Transcatheter Aortic Valve Replacement: A Meta-Analysis and Modeling Study. J. Am. Coll. Cardiol..

[2] Carabello B.A., Paulus W.J. (2009). Aortic stenosis. Lancet.

[3] Iung B., Vahanian A. (2011). Epidemiology of valvular heart disease in the adult. Nat. Rev. Cardiol..

[4] Holmes D.R., Brennan J.M., Rumsfeld J.S., Dai D., O’brien S.M., Vemulapalli S., Edwards F.H., Carroll J., Shahian D., Grover F. (2015). Clinical Outcomes at 1 Year Following Transcatheter Aortic Valve Replacement. JAMA.

[5] Barili F., Pacini D., Capo A., Rasovic O., Grossi C., Alamanni F., Di Bartolomeo R., Parolari A. (2013). Does EuroSCORE II perform better than its original versions? A multicentre validation study. Eur. Heart J..

[6] Osnabrugge R.L., Speir A.M., Head S.J., Fonner C.E., Fonner E., Kappetein A.P., Rich J.B. (2014). Performance of EuroSCORE II in a large US database: Implications for transcatheter aortic valve implantation. Eur. J. Cardio-Thorac. Surg..

[7] Kumar A., Sato K., Narayanswami J., Banerjee K., Andress K., Lokhande C., Mohananey D., Anumandla A.K., Khan A.R., Sawant A.C. (2018). Current Society of Thoracic Surgeons Model Reclassifies Mortality Risk in Patients Undergoing Transcatheter Aortic Valve Replacement. Circ. Cardiovasc. Interv..

[8] Thyregod H.G.H., Steinbrüchel D.A., Ihlemann N., Nissen H., Kjeldsen B.J., Petursson P., Chang Y., Franzen O.W., Engstrøm T., Clemmensen P. (2015). Transcatheter Versus Surgical Aortic Valve Replacement in Patients with Severe Aortic Valve Stenosis. J. Am. Coll. Cardiol..

[9] Xue Q.-L. (2011). The Frailty Syndrome: Definition and Natural History. Clin. Geriatr. Med..

[10] Shi S., Afilalo J., Lipsitz L.A., Popma J.J., Khabbaz K.R., Laham R.J., Guibone K., Grodstein F., Lux E., Kim D.H. (2018). Frailty Phenotype and Deficit Accumulation Frailty Index in Predicting Recovery after Transcatheter and Surgical Aortic Valve Replacement. J. Gerontol. Ser. A.

[11] Shimura T., Yamamoto M., Kano S., Kagase A., Kodama A., Koyama Y., Tsuchikane E., Suzuki T., Otsuka T., Kohsaka S. (2017). Impact of the Clinical Frailty Scale on Outcomes after Transcatheter Aortic Valve Replacement. Circulation.

[12] Kleczynski P., Dziewierz A., Bagienski M., Rzeszutko L., Sorysz D., Trebacz J., Sobczynski R., Tomala M., Stapor M., Dudek D. (2017). Impact of frailty on mortality after transcatheter aortic valve implantation. Am. Hearth J..

[13] Puls M., Sobisiak B., Bleckmann A., Jacobshagen C., Danner B.C., Hünlich M., Beißbarth T., Schöndube F., Hasenfuß G., Seipelt R. (2014). Impact of frailty on short- and long-term morbidity and mortality after transcatheter aortic valve implantation: Risk assessment by Katz Index of activities of daily living. Eurointervention.

[14] Strom J.B., Xu J., Orkaby A.R., Shen C., Song Y., Charest B.R., Kim D.H., Cohen D.J., Kramer D.B., Spertus J.A. (2021). Role of Frailty in Identifying Benefit from Transcatheter Versus Surgical Aortic Valve Replacement. Circ. Cardiovasc. Qual. Outcomes.

[15] Sá M.P., Erten O., Ramlawi B. (2022). Transcatheter Aortic Valve Implantation in Elderly Patients with Aortic Valve Stenosis: The Role of Frailty, Malnutrition, and Sarcopenia. J. Am. Heart Assoc..

[16] Bs Q.P.H., Maltagliati A.J., Shi S.M., Afilalo J., Popma J.J., Khabbaz K.R., Laham R.J., Np K.G., Kim D.H. (2019). A Practical Two-Stage Frailty Assessment for Older Adults Undergoing Aortic Valve Replacement. J. Am. Geriatr. Soc..

[17] Green P., Woglom A.E., Genereux P., Daneault B., Paradis J.-M., Schnell S., Hawkey M., Maurer M.S., Kirtane A.J., Kodali S. (2012). The Impact of Frailty Status on Survival after Transcatheter Aortic Valve Replacement in Older Adults with Severe Aortic Stenosis. JACC Cardiovasc. Interv..

[18] Anand A., Harley C., Visvanathan A., Shah A.S.V., Cowell J., MacLullich A., Shenkin S., Mills N.L. (2017). The relationship between preoperative frailty and outcomes following transcatheter aortic valve implantation: A systematic review and meta-analysis. Eur. Heart. J. Qual. Care Clin. Outcomes.

[19] Senior Health Calculator: Online Tool for Providers|BIDMC of Boston. https://www.bidmc.org/research/research-by-department/medicine/gerontology/calculator.

[20] Kim D.H., Glynn R.J., Avorn J., Lipsitz L.A., Rockwood K., Pawar A., Schneeweiss S. (2018). Validation of a Claims-Based Frailty Index Against Physical Performance and Adverse Health Outcomes in the Health and Retirement Study. J. Gerontol. Ser. A.

[21] Risk Calculator|STS. https://www.sts.org/resources/risk-calculator.

[22] EuroScore Website—Calculator. https://www.euroscore.org/index.php?id=17.

[23] Puls M., Sobisiak B., Jacobshagen C., Danner B., Schoendube F., Hasenfuss G., Seipelt R., Schillinger W. (2013). Katz-Index effectively predicts long-term mortality after Transcatheter Aortic Valve Implantation (TAVI). Eur. Heart J..

[24] Kukull W., Larson E., Teri L., Bowen J., McCormick W., Pfanschmidt M. (1994). The mini-mental state examination score and the clinical diagnosis of dementia. J. Clin. Epidemiol..

[25] Kappetein A.P., Head S.J., Généreux P., Piazza N., van Mieghem N.M., Blackstone E.H., Brott T.G., Cohen D.J., Cutlip D.E., van Es G.-A. (2012). Updated standardized endpoint definitions for transcatheter aortic valve implantation: The Valve Academic Research Consortium-2 consensus document†. Eur. Heart J..

[26] Javed A.A., Ma J., Anderson L.N., Mayhew A.J., So H.Y., Griffith L.E., Gilsing A., Raina P. (2022). Age-appropriate BMI cut-points for cardiometabolic health risk: A cross-sectional analysis of the Canadian Longitudinal Study on Aging. Int. J. Obes..

[27] Dias F.L.D.C., Teixeira A.L., Guimarães H.C., Barbosa M.T., Resende E.D.P.F., Beato R.G., Carmona K.C., Caramelli P. (2017). Accuracy of the 15-item Geriatric Depression Scale (GDS-15) in a community-dwelling oldest-old sample: The Pietà Study. Trends Psychiatry Psychother..

[28] Bautmans I., Onyema O., Van Puyvelde K., Pleck S., Mets T. (2011). Grip work estimation during sustained maximal contraction: Validity and relationship with dependency and inflammation in elderly persons. J. Nutr. Health Aging.

[29] Cruz-Jentoft A.J., Bahat G., Bauer J., Boirie Y., Bruyère O., Cederholm T., Cooper C., Landi F., Rolland Y., Sayer A.A. (2019). Sarcopenia: Revised European consensus on definition and diagnosis. Age Ageing.

[30] Buatois S., Perret-Guillaume C., Gueguen R., Miget P., Vançon G., Perrin P., Benetos A. (2010). A Simple Clinical Scale to Stratify Risk of Recurrent Falls in Community-Dwelling Adults Aged 65 Years and Older. Phys. Ther..

[31] Guralnik J.M., Simonsick E.M., Ferrucci L., Glynn R.J., Berkman L.F., Blazer D.G., Scherr P.A., Wallace R.B. (1994). A Short Physical Performance Battery Assessing Lower Extremity Function: Association with Self-Reported Disability and Prediction of Mortality and Nursing Home Admission. J. Gerontol..

[32] Tinetti M.E., Speechley M., Ginter S.F. (1988). Risk Factors for Falls among Elderly Persons Living in the Community. N. Engl. J. Med..

[33] Podsiadlo D., Richardson S. (1991). The Timed “Up & Go”: A Test of Basic Functional Mobility for Frail Elderly Persons. J. Am. Geriatr. Soc..

[34] Charlson M., Szatrowski T.P., Peterson J., Gold J. (1994). Validation of a combined comorbidity index. J. Clin. Epidemiol..

[35] Chang S.S., Weiss C.O., Xue Q.-L., Fried L.P. (2012). Association between inflammatory-related disease burden and frailty: Results from the Women’s Health and Aging Studies (WHAS) I and II. Arch. Gerontol. Geriatr..

[36] Drudi L.M., Ades M., Turkdogan S., Huynh C., Lauck S., Webb J.G., Piazza N., Martucci G., Langlois Y., Perrault L.P. (2018). Association of Depression with Mortality in Older Adults Undergoing Transcatheter or Surgical Aortic Valve Replacement. JAMA Cardiol..

[37] Kagansky N., Berner Y., Koren-Morag N., Perelman L., Knobler H., Levy S. (2005). Poor nutritional habits are predictors of poor outcome in very old hospitalized patients. Am. J. Clin. Nutr..

[38] Bansal A., Gupta S., Aggarwal M., Jain V., Gad M.M., Verma B.R., Kapadia S.R. (2020). Impact of Malnutrition on Outcomes among Patients Undergoing Transcatheter Aortic Valve Implantation. Am. J. Cardiol..

[39] Schoenenberger A.W., Stortecky S., Neumann S., Moser A., Jüni P., Carrel T., Huber C., Gandon M., Bischoff S., Schoenenberger C.-M. (2012). Predictors of functional decline in elderly patients undergoing transcatheter aortic valve implantation (TAVI). Eur. Heart J..

[40] Lindman B.R., Alexander K.P., O’Gara P.T., Afilalo J. (2014). Futility, Benefit, and Transcatheter Aortic Valve Replacement. JACC Cardiovasc. Interv..

[41] Shimura T., Yamamoto M., Kano S., Sago M., Tsunaki T., Kagase A., Koyama Y., Tsujimoto S., Otsuka T., Yashima F. (2020). Predictors and Prognostic Impact of Nutritional Changes After Transcatheter Aortic Valve Replacement. Cardiovasc. Revascularization Med..

[42] Vahanian A., Beyersdorf F., Praz F., Milojevic M., Baldus S., Bauersachs J., Capodanno D., Conradi L., De Bonis M., De Paulis R. (2021). 2021 ESC/EACTS Guidelines for the management of valvular heart disease. Eur. Heart J..

[43] Kim D.H., Afilalo J., Shi S.M., Popma J.J., Khabbaz K.R., Laham R.J., Grodstein F., Guibone K., Lux E., Lipsitz L.A. (2019). Evaluation of Changes in Functional Status in the Year after Aortic Valve Replacement. JAMA Intern. Med..

[44] Ribeiro G.S., Melo R.D., Deresz L.F., Lago P.D., Pontes M.R., Karsten M. (2017). Cardiac rehabilitation programme after transcatheter aortic valve implantation versus surgical aortic valve replacement: Systematic review and meta-analysis. Eur. J. Prev. Cardiol..

[45] Reber E., Gomes F., Vasiloglou M.F., Schuetz P., Stanga Z. (2019). Nutritional Risk Screening and Assessment. J. Clin. Med..

