# Supplementary material for: Depression and Malnutrition for Prediction of Mortality after Transcatheter Aortic Valve Replacement: A Registry Study of a Tertiary Referral Hospital
Source: Diagnostics (Basel). 2023 Aug 1;13(15):2561. doi: 10.3390/diagnostics13152561 (PMC10416912; doi:10.3390/diagnostics13152561)

Supplemental Table S1 Baseline characteristics according to depression status

	**At risk for depression (n=18)**	**Not at risk for depression (n=73)**	**P-value**
Clinical			
Male, n (%)	8 (44)	28 (38)	0.636
Age (years)	85 ± 4	84 ± 4	0.705
Body mass index (kg/m^2)	26.3 ± 5.1	27.8 ± 4.8	0.237
Hypertension, n (%)	17 (94)	55 (75)	0.074
Diabetes mellitus, n (%)	5 (28)	17 (23)	0.690
Atrial fibrillation, n (%)	8 (44)	25 (34)	0.420
Prior MI, n (%)	3 (17)	11 (15)	0.866
CAD, n (%)	7 (39)	31 (42)	0.783
Prior stroke or TIA, n (%)	0 (0)	8 (11)	0.141
Sternotomy, n (%)	3 (17)	20 (27)	0.348
COPD, n (%)	4 (22)	13 (18)	0.667
EuroSCORE II	4.8 (3.0-7.8)	4.6 (2.9-7.9)	0.750
STS-PROM	4.0 (2.7-5.2)	3.9 (2.4-5.0)	0.746
Medication, n (%)			
ADP inhibitor	0 (0)	6 (8)	0.208
ACE/ARB inhibitor	12 (67)	43 (59)	0.546
Antidepressants	2 (11)	5 (7)	0.543
Hemodynamic and laboratory characteristics			
AVA (cm²)	0.72 ± 0.13	0.77 ± 0.24	0.250
PG (mmHg)	61.2 ± 19.4	73.8 ± 29.6	0.091
LVEF (%)	50 (44-56)	55 (50-60)	**0.041**
TAPSE (mm)	16.5 ± 5.7	20.0 ± 4.9	**0.042**
SPAP (mmHg)	47.1 ± 23.7	41.1 ± 12.9	0.342
LAVI (mL/m^2)	55.6 ± 21.6	49.7 ± 13.9	0.266
eGFR (mL/min/1.73m²)	57.2 ± 11.9	52.6 ± 13.8	0.201
Hematocrit (%)	35.3 ± 12.4	34.0 ± 12.0	0.669
Albumin (g/L)	40.3 ± 10.0	39.6 ± 4.1	0.800
Geriatric characteristics			
Katz <7, n (%)	12 (67)	38 (52)	0.264
MMSE <27, n (%)	10 (56)	30 (41)	0.268
Polypharmacy, n (%)	15 (83)	69 (94)	0.111
Timed up and go ≥20 sec, n (%)	2 (12)	14 (20)	0.501
Chair stand test ≥14 sec, n (%)	9 (69)	53 (75)	0.683
Gait speed ≤0.8 m/sec, n (%)	8 (80)	21 (34)	**0.006**
Tinetti <20, n (%)	2 (15)	13 (21)	0.665
SPPB <10, n (%)	8 (73)	44 (68)	0.740
Grip strength <71 kPa (men) & <42 kPa (women), n (%)	7 (78)	38 (67)	0.506
CCI	5.1 ± 0.9	5.0 ± 1.1	0.804
CGI-FI >0.2, n (%)	3 (17)	9 (12)	0.626
Malnutrition, n (%)	3 (23)	7 (11)	0.235
Procedural and hospitalization characteristics			
Full anesthesia, n (%)	16 (89)	51 (70)	0.101

TF approach, n (%)	17 (94)	72 (99)	0.278
Length of stay (days)	10 (9-20)	8 (6-12)	**0.004**
Pacemaker, n (%)	3 (17)	14 (19)	0.807
MR ≥ 2/4, n (%)	3 (17)	25 (34)	0.148
AR ≥ 2/4, n (%)	3 (18)	7 (10)	0.375
Geriatric follow-up, n (%)	6 (33)	27 (37)	0.773

Data are presented as number (percent), mean ± standard deviation if normally distributed or median (interquartile range) if not normally distributed.

MI: myocardial infarction; CAD: coronary artery disease; TIA: transient ischemic attack; COPD: chronic obstructive pulmonary disease; STS-PROM: Society of Thoracic Surgery predicted risk of mortality score, ADP: adenosine diphosphate; ACE: angiotensin converting enzyme; ARB: angiotensin receptor blocker; AVA: aortic valve area; PG: peak gradient; LVEF: left ventricular ejection fraction; TAPSE: tricuspid annular plane systolic excursion; SPAP: systolic pulmonary artery pressure; LAVI: left atrium volume index; eGFR: estimated glomerular filtration rate; MMSE: mini mental state examination; SPPB: short physical performance battery; kPa: kilopascal; CCI: Charlson comorbidity index; CGA-FI: comprehensive geriatric assessment frailty index; TF: transfemoral; MR: mitral regurgitation; AR: aortic regurgitation.

Supplemental Table S2 Baseline characteristics according to malnutrition status

	**Malnutrition (n=11)**	**No malnutrition (n=69)**	**P-value**
Clinical			
Male, n (%)	6 (54)	25 (36)	0.247
Age (years)	83 ± 5	84 ± 4	0.309
Body mass index (kg/m^2)	26.7 ± 7.3	27.8 ± 4.8	0.643
Hypertension, n (%)	8 (73)	52 (75)	0.851
Diabetes mellitus, n (%)	4 (36)	15 (22)	0.290
Atrial fibrillation, n (%)	4 (36)	26 (38)	0.933
Prior MI, n (%)	1 (9)	11 (16)	0.555
CAD, n (%)	4 (36)	28 (41)	0.791
Prior stroke or TIA, n (%)	2 (18)	6 (9)	0.330
Sternotomy, n (%)	2 (18)	15 (22)	0.789
COPD, n (%)	2 (18)	13 (19)	0.959
EuroSCORE II	7.4 (2.5-10.2)	4.3 (2.5-6.5)	0.295
STS-PROM	3.3 (2.5-4.2)	3.9 (2.6-5.0)	0.446
Medication, n (%)			
ADP inhibitor	0 (0)	7 (10)	0.269
ACE/ARB inhibitor	7 (64)	40 (58)	0.723
Antidepressants	1 (9)	4 (6)	0.675
Hemodynamic and laboratory characteristics			
AVA (cm^2)	0.80 ± 0.23	0.76 ± 0.22	0.600
PG (mmHg)	70.1 ± 24.9	70.9 ± 28.2	0.932
LVEF (%)	50 (30-55)	55 (50-60)	**0.049**
TAPSE (mm)	20.0 ± 7.3	19.9 ± 5.1	0.939
SPAP (mmHg)	49.1 ± 13.7	42.0 ± 16.4	0.204
LAVI (mL/m^2)	60.1 ± 22.0	48.1 ± 13.4	**0.034**
EGFR (mL/min/1.73m^2)	51.1 ± 10.3	54.4 ± 14.3	0.465
Hematocrit (%)	29.2 ± 14.3	34.3 ± 12.2	0.211
Geriatric characteristics			
Katz <7, n (%)	4 (36)	38 (56)	0.229
MMSE <27, n (%)	3 (27)	31 (45)	0.271
Polypharmacy, n (%)	11 (100)	63 (91)	0.309
Timed up and go ≥20 sec, n (%)	3 (33)	12 (18)	0.275
Chair stand test ≥14 sec, n (%)	5 (62)	48 (74)	0.497
Gait speed ≤0.8 m/sec, n (%)	2 (40)	23 (40)	0.988
Tinetti <20, n (%)	2 (25)	12 (21)	0.780
SPPB <10, n (%)	6 (75)	37 (65)	0.572
Grip strength <71 kPa (men) & <42 kPa (women), n (%)	3 (75)	36 (69)	0.809
CCI	5.4 ± 0.9	4.9 ± 1.1	0.232
CGI-FI >0.2, n (%)	1 (9)	9 (13)	0.713
GDS-15 ≥5, n (%)	3 (30)	10 (15)	0.235
Procedural and hospitalization characteristics			
Full anesthesia, n (%)	9 (82)	48 (70)	0.404

TF approach, n (%)	10 (91)	68 (97)	0.132
Length of stay (days)	15 (7-27)	8 (6-12)	**0.020**
Pacemaker, n (%)	3 (27)	12 (17)	0.436
MR ≥ 2/4, n (%)	2 (18)	24 (35)	0.275
AR ≥ 2/4, n (%)	1 (9)	8 (12)	0.772
Geriatric follow-up, n (%)	4 (36)	25 (36)	0.993

Data are presented as number (percent), mean ± standard deviation if normally distributed or median (interquartile range) if not normally distributed.

MI: myocardial infarction; CAD: coronary artery disease; TIA: transient ischemic attack; COPD: chronic obstructive pulmonary disease; STS-PROM: Society of Thoracic Surgery predicted risk of mortality score, ADP: adenosine diphosphate; ACE: angiotensin converting enzyme; ARB: angiotensin receptor blocker; AVA: aortic valve area; PG: peak gradient; LVEF: left ventricular ejection fraction; TAPSE: tricuspid annular plane systolic excursion; SPAP: systolic pulmonary artery pressure; LAVI: left atrium volume index; eGFR: estimated glomerular filtration rate; MMSE: mini mental state examination; SPPB: short physical performance battery; kPa: kilopascal; CCI: Charlson comorbidity index; CGA-FI: comprehensive geriatric assessment frailty index; GDS-15: geriatric depression scale 15; TF: transfemoral; MR: mitral regurgitation; AR: aortic regurgitation.

Supplemental Table S3 Univariable Cox regression analysis of frailty markers for all-cause mortality prediction

	**HR**	**95% CI**	**P-value**
Katz	1.059	0.464-2.416	0.892
MMSE	0.758	0.328-1.752	0.517
Polypharmacy	0.844	0.198-3.603	0.819
Timed up and go	1.052	0.356-3.112	0.927
Chair stand test	1.291	0.469-3.555	0.621
Gait speed	1.066	0.406-2.802	0.897
Tinetti	1.199	0.394-3.646	0.750
SPPB	1.374	0.490-3.856	0.546
Grip strength	0.628	0.223-1.764	0.377
GDS-15	1.203	1.057-1.370	**0.005**
Malnutrition	3.672	1.443-9.345	**0.006**
CCI	1.090	0.773-1.535	0.623
CGA-FI	0.958	0.284-3.227	0.945

HR: hazard ratio; CI: confidence interval; MMSE: mini mental state examination; SPPB: short physical performance battery; GDS-15: geriatric depression scale 15; CCI: Charlson comorbidity index; CGA-FI: comprehensive geriatric assessment frailty index.

Supplemental Figure S1. Flow chart of study population after applying exclusion and inclusion criteria.

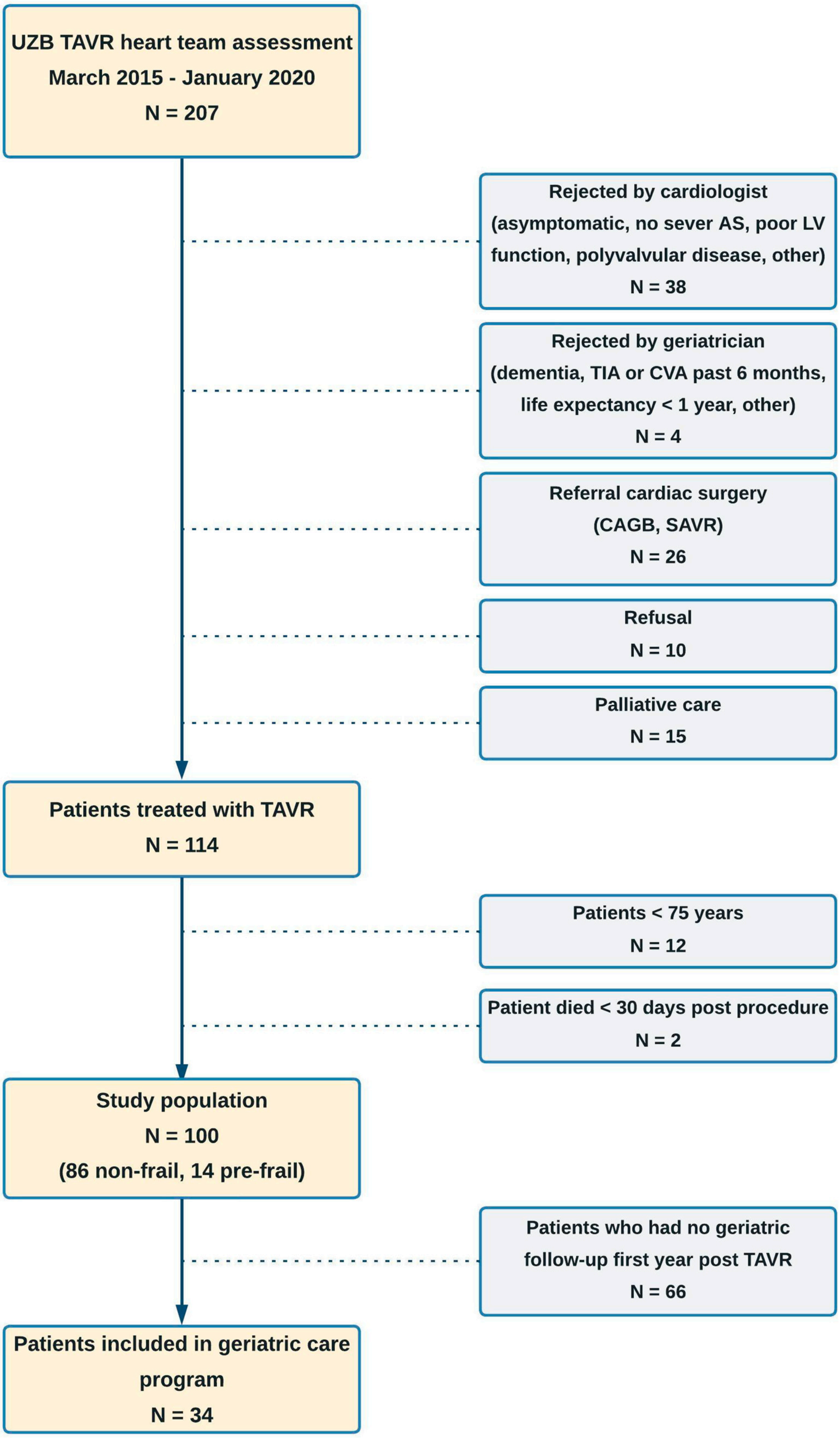

Supplemental figure S2. Kaplan-Meier curves with log-rank test for overall survival of patients treated with TAVR according to inclusion in post TAVR geriatric care program.

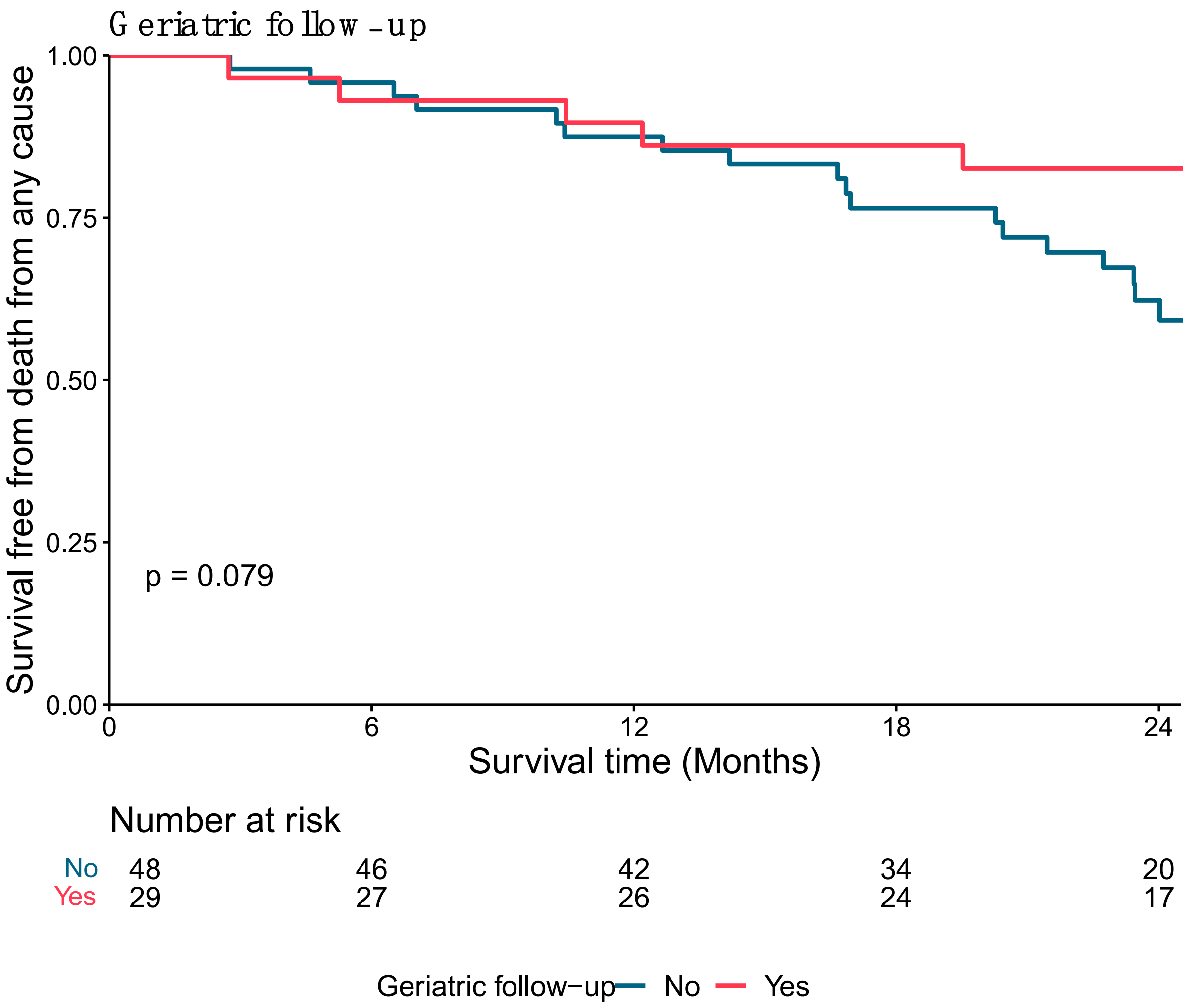

Supplement: Supplementary file 1 [file diagnostics-13-02561-s001.zip › diagnostics-2502510-supplementary.pdf]
